# Prevalence of strongyloidiasis in immunocompromised patients in Mazandaran province of northern Iran: A comprehensive study utilizing simultaneous parasitological, serological, and molecular techniques

**DOI:** 10.1016/j.parepi.2025.e00425

**Published:** 2025-04-25

**Authors:** Reza Saberi, Aliasghar Ghorbanzadeh, Rabeeh Tabaripour, Shahabeddin Sarvi, Shirzad Gholami, Seyed Abdollah Hosseini

**Affiliations:** a Toxoplasmosis Research Center, Communicable Diseases Institute, School of Medicine, Mazandaran University of Medical Sciences, Sari, Iran; b Department of Medical Parasitology and Mycology, School of Medicine, Shahid Beheshti University of Medical Sciences, Tehran, Iran; c Student Research Committee, Mazandaran University of Medical Sciences, Sari, Iran; d Department of Parasitology, School of Medicine, Mazandaran University of Medical Sciences, Sari, Iran

**Keywords:** Strongyloidiasis, Immunodeficiency, ELISA, PCR, Mazandaran

## Abstract

**Introduction:**

*Strongyloides stercoralis* is a soil-transmitted helminth (STH) responsible for strongyloidiasis, a neglected tropical disease (NTD) that affects nearly 614 million people globally. This intestinal nematode poses significant health risks, particularly in immunocompromised individuals. The present study aimed to investigate the prevalence of *S. stercoralis* in high-risk populations in northern Iran, employing a combination of parasitological, serological, and molecular techniques.

**Methods:**

Blood and fecal samples were collected from 92 patients in Mazandaran province, northern Iran, consisting of 52 patients with HIV+/AIDS and 40 cancer patients undergoing chemotherapy or corticosteroid treatment. Initially, all fecal samples were examined using the nutrient agar culture method for parasitological assessment. Following this, DNA extraction was performed on all samples for identify *S. stercoralis* (by COX1- Nested PCR). Additionally, the sera of the patients were analyzed using the enzyme-linked immunosorbent assay (ELISA) kit (NovaTec Immunodiagnostica GmbH, Dietzenbach, Germany).

**Results:**

The stool samples from these patients were negative in agar plate cultures. Among the 92 patients in the study, stool microscopy for *Strongyloides* rhabditiform larvae was positive in three cases. Using nested PCR, four samples (4.34 %) tested positive for *S. stercoralis*. Serological investigations revealed that 4 out of 52 HIV-positive patients (7.69 %) and 15 out of 40 cancer patients (37.5 %) had a history of infection with *S. stercoralis*.

**Conclusions:**

These results emphasis the importance of employing a multifaceted diagnostic approach, combining parasitological, serological, and molecular techniques, to accurately identify infections in at risk populations. Given the potential for severe complications associated with strongyloidiasis in immunocompromised individuals, regular screening and prompt treatment are essential to reduce health risks.

## 1 Introduction

Strongyloidiasis is a significant soil-transmitted helminthiasis (STH) primarily caused by the nematode *Strongyloides stercoralis* (*S. stercoralis*) (Varatharajalu and Rao, 2016). This intestinal parasite is classified as one of the neglected tropical diseases (NTDs), affecting nearly 614 million individuals worldwide (Buonfrate et al., 2020). The infection is predominantly found in tropical and subtropical regions (Puthiyakunnon et al., 2014). Clinically, strongyloidiasis can present with a wide spectrum of symptoms (Toledo et al., 2015). In immunocompetent individuals, infections may be mild or asymptomatic, while in immunocompromised patients such as those with HIV or undergoing chemotherapy, the disease can lead to severe complications like hyper infection syndrome or disseminated strongyloidiasis (Toledo et al., 2015; Marcos et al., 2011).

The diagnosis of *S. stercoralis* infection typically relies on parasitological methods such as direct smear examination and agar plate culture; however, these techniques often exhibit low sensitivity and may yield false-negative results (Ericsson et al., 2001). Serological tests provide an alternative but can overestimate disease prevalence due to persistent antibody responses even after successful treatment (Buonfrate et al., 2015). Molecular techniques have emerged as valuable tools for accurate detection, allowing for rapid identification of *S. stercoralis* (Chan and Thaenkham, 2023; Buonfrate et al., 2022).

Given the complexities associated with its life cycle and the potential for severe disease in at risk populations, identification of *S. stercoralis* is crucial for improving public health strategies aimed at prevention and control of strongyloidiasis (Chan et al., 2023). This study aims to further investigate the prevalence of *S. stercoralis* in high-risk groups using a combination of parasitological, serological, and molecular techniques in northern Iran, where environmental conditions favor its transmission and where immune compromised individuals are at heightened risk for severe outcomes.

## 2 Material and methods

### 2.1 Ethical statements

The ethics committee of the Mazandaran University of Medical Sciences approved the research (IR.MAZUMS.REC.1401.112). The project was financially supported by the Toxoplasmosis Research Center, Mazandaran University of Medical Sciences, Sari, Iran (Grant no. 13734). The patients were informed about the study, and written consent was obtained from all participants. Demographic and clinical data on human isolates were recorded in a questionnaire.

### 2.2 Study area

**Fig. 1:**
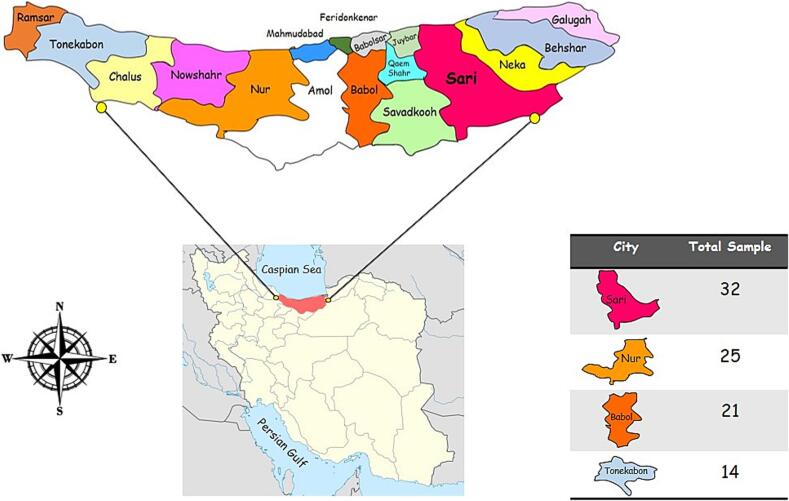
Geographical location of Mazandaran province, Iran.

The present cross-sectional study was conducted in Mazandaran province, located in the northern region of Iran (latitude: 35°47ʹ to 36°35ʹN, longitude: 50°34ʹ to 54°10ʹE). Mazandaran province including 19 cities and has a population of approximately 3,073,943 individuals. This area is characterized by a subtropical climate, with an average annual relative humidity of 83 %, an average temperature of 18 °C, and rainfall occurring throughout all four seasons of the year (Fig. 1).

### 2.3 Study population

The target population for this study consists of 92 immunocompromised patients, including HIV-positive/AIDS patients (*n* = 52) and cancer patients undergoing corticosteroid treatment or chemotherapy (*n* = 40) who were referred to Imam Sajjad Hospital, Shahid Rajaei, Sari Health Center, and Amirkola Children's Hospital were included in the study. Among the participants, 38 patients (41.3 %) were male, while 54 patients (58.7 %) were female. It should be noted the HIV-positive patients were confirmed through the Western blot test. Additionally, cancer patients undergoing chemotherapy were diagnosed using serological, imaging, and pathology tests.

The inclusion and exclusion criteria for participants were as follows: Inclusion Criteria: 1) HIV-positive individuals confirmed by the Western blot test and had a CD4 count below 500 cells/μL, 2) Cancer patients undergoing chemotherapy who diagnosis was validated through serological, imaging, and pathology assessments.

Exclusion Criteria: 1) Patients who did not provide informed consent, 2) Cancer patients who received less than four rounds of chemotherapy, and 3) HIV positive people with CD4 count above 500 cells/μL.

### 2.4 Sample collection

For each patient participating in this research study, biological samples, including blood and stool, were collected. A venous blood sample of 5 ml was obtained from each subject and promptly transported to the laboratory at the Department of Parasitology, School of Medicine, Mazandaran University of Medical Sciences. Following collection, serum was separated through centrifugation at 335*g* for 10 min and stored at −20 °C until the ELISA test was conducted. Each participant was provided with a stool collection tube and was instructed to collect a fresh fecal specimen.

### 2.5 Parasitological methods

Stool samples were processed using the formalin-ether concentration technique. The sediment obtained from these concentrated samples was first examined under a 10× magnification lens, followed by a detailed examination at 40× magnification.

For the culture method, approximately 3–4 g of fresh stool sample were inoculated onto a nutrient agar plate and incubated for 3–4 days at a temperature of 28–30 °C. After incubation, the plates were carefully inspected using a stereomicroscope to identify any larvae present. To collect the larvae, the surface of the agar plate was washed with a physiological saline solution and then centrifuged at 1000 ×*g* for 2 min. The sediment obtained from this process was fixed in 10 % formalin for identification purposes. Positive samples of *Strongyloides* spp. was preserved in 70 % ethanol at 4 °C for further molecular examinations.

### 2.6 Serological methods

An enzyme-linked immunosorbent assay (ELISA) was performed using an IgG ELISA kit (NovaTec Immunodiagnostica GmbH, Dietzenbach, Germany) with recombinant immunodiagnostic antigen (NIE), following the manufacturer's instructions. Absorbance values were recorded at 450 nm using a Dynex DS2® automated ELISA reader. The results were expressed in NovaTec units (NTU), with a cutoff value established at 10 NTU. Samples yielding values greater than 11 NTU were classified as positive, while those between 9 and 11 NTU were deemed equivocal, and values below 9 NTU were considered negative.

### 2.7 Molecular methods

DNA extraction from stool samples was performed using the Stool DNA Extraction Mini Kit (Favorgen Biotech Corporation, Taiwan), following the manufacturer's instructions. Two sets of primer pairs were utilized for nested PCR to amplify a 509-bp target in the first PCR round and a 261-bp target in the second round, specifically targeting the mitochondrial cytochrome *c* oxidase subunit 1 (cox1) gene. The primers used for the first amplification round were EF (5′-TGGTTTGGGTACTAGTTG-3′) and ER (5′-ATGAGCTCAAACTACACA-3′). For the second amplification round, the primers IF (5′-TTCTAGTGTTGATTTGGC-3′) and IR (5′-TTACCACCAAAACTAGGATC-3′) were employed.

The cycling conditions for the first round consisted of an initial denaturation step at 95 °C for 5 min, followed by 35 cycles of denaturation at 95 °C for 45 s, annealing at 55 °C for 60 s, and extension at 72 °C for 1 min, concluding with a final extension at 72 °C for 5 min. For the second amplification round, the cycling conditions included an initial denaturation at 95 °C for 2 min, followed by 32 cycles of denaturation at 94 °C for 30 s, annealing at 60 °C for 1 min, and extension at 72 °C for 45 s, with a final extension at 72 °C for 5 min. Finally, a 5-μL aliquot of the nested PCR product was then subjected to electrophoresis on a 1.5 % agarose gel to visualize the amplified DNA fragments.

### 2.8 Sequencing and phylogenetic analysis

PCR products of the cytochrome *c* oxidase subunit 1 (Cox1) gene from four distinct *Strongyloides* isolates were purified and sequenced using an ABI Prism™ 3730 Genetic Analyzer (Applied Biosystems, Foster City, California, USA) through the services of Macrogen Company (Seoul, South Korea). Multiple sequence alignments were conducted using BioEdit version 7.0.5 and analyzed with MEGA x software. To construct a phylogenetic tree for the *Strongyloides* datasets, we employed maximum likelihood (ML) methods based on the Kimura 2-parameter model in MEGA version x. Additionally, bootstrap resampling analysis with 1000 replications was performed to evaluate the confidence of the branches within each phylogenetic tree (Kumar et al., 2016). The sequence of *Strongyloides planiceps* with GenBank accession number AB526296.1was utilized as an out group in the phylogenetic analysis.

### 2.9 Statistical analysis

In this study, the Chi-square test was utilized to assess potential disparities in the prevalence of *S. stercoralis* across the various examined variables. Statistical analysis was conducted using SPSS (Version 20), with a *P*-value of less than 0.05 considered indicative of statistical significance.

## 3 Results

### 3.1 Direct microscopy identification

Microscopic examination for the detection of rhabditiform first stage larvae was conducted, revealing the presence of larvae in three stool samples, which corresponds to a prevalence of 3.26 %. However, it is noteworthy that the stool samples from these patients tested negative in agar plate cultures.

### 3.2 Serological identification and risk factor results

The seroprevalence of strongyloidiasis among the participants was found to be 20.65 % (19 out of 92). Notably, the seroprevalence of *S. stercoralis* was higher in males (23.68 %) compared to females (18.52 %), although this difference was not statistically significant (*P* = 0.55). Among those above 50 years, the infection rate with *S. stercoralis* was 19.64 %, compared to 22.22 % in those below 51 years; however, no statistically significant differences were observed between age and *S. stercoralis* infection (*P* = 0.76). A significant portion of participants were illiterate (41.3 %), and this group exhibited the highest seroprevalence of strongyloidiasis at 31.58 %. Among the participants, 39.13 % resided in urban areas and 60.87 % in rural areas. The infection rate of *S. stercoralis* was significantly higher in rural areas (28.57 %) compared to urban areas (8.33 %) (*P* = 0.04 and *P* = 0.01, respectively). Most seropositive patients reported experiencing abdominal pain (22.45 %) and diarrhea (21.95 %); however, no statistically significant differences were observed for these symptoms (*P* = 0.65 and *P* = 0.78, respectively). The results indicated that 7.69 % of HIV-positive patients (4 out of 52) and 37.5 % of cancer patients (15 out of 40) tested positive for anti-*S. stercoralis* antibodies. This finding reveals a significant association between disease type and *S. stercoralis* infection (*P* = 0.0005) (Table. 1).

**Table 1:** Analysis of microscopic, serological, and molecular results of Strongyloidiasis in immunocompromised patients in Mazandaran province of northern Iran based on demographic factors.

Variables		Sample size	ELISA No. (%)	*P* value	nPCR No. (%)	Microscopy No. (%)
Groups	HIV+	52	4 (7.69)	0.005	2 (3.85)	2 (3.85)
	Cancer	40	15 (37.5)		2 (5)	1 (2.5)
Sex	Male	38	10 (18.52)	0.55	3 (7.89)	2 (5.26)
	Female	54	9 (23.68)		1 (1.85)	1 (1.85)
Age	>50	56	11 (19.64)	0.76	2 (3.57)	1 (1.78)
	<50	36	8 (22.22)		2 (5.56)	2 (5.56)
Education	Illiterate	38	12 (31.58)	0.04	3 (8.33)	2 (5.26)
	Undergraduate	32	6 (18.75)		1 (3.12)	1 (3.12)
	Diploma and university	22	1 (4.54)		0 (0)	0 (0)
Residence	Rural	56	16 (28.57)	0.01	3 (5.36)	2 (3.57)
	Urban	36	3 (8.33)		1 (2.78)	1 (2.78)
Abdominal pain	Yes	49	11 (22.45)	0.65	3 (6.12)	2 (4.08)
	No	43	8 (18.6)		1 (2.32)	1 (2.32)
Diarrhea	Yes	41	9 (21.95)	0.78	2 (4.88)	2 (4.88)
	No	51	10 (19.6)		2 (3.92)	1 (1.96)

### 3.3 Molecular analysis

**Fig. 2:**
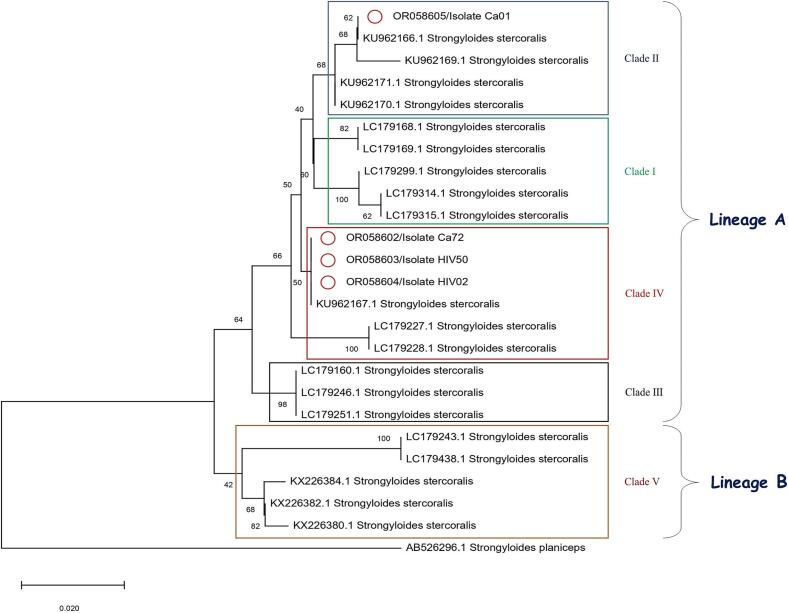
Phylogenetic tree of sequences of *S. stercoralis* in the present study and other *S. stercoralis* sequences obtained from GenBank. Maximum likelihood tree of LRVs built on completed sequence alignment using Tamura 3-parameter models.

All fecal samples were subjected to COX1-Nested PCR for identification. The PCR analysis yielded an amplified fragment of approximately 261 bp in four samples (4.35 %), indicating the presence of *S. stercoralis* DNA. Among these, two cases (5 %) were molecularly positive in the cancer group, while two positive cases (3.85 %) were identified in the HIV positive/AIDS group. Among the four individuals who tested positive by nested PCR, all were serologically positive, and three were also microscopically positive. Table 2 presents the clinical and demographic characteristics of positive strongyloidiasis cases confirmed by microscopic, serological, and molecular methods. To enhance the reliability of our findings, four samples were sequenced and deposited in GenBank (Accession numbers: OR058602–OR058605). These sequences exhibited 98–100 % similarity with existing *S. stercoralis* sequences available in GenBank. A maximum likelihood phylogenetic tree was constructed based on the sequences of the current *S. stercoralis* isolates and those available in GenBank (Fig. 2). The current isolate clustered within Clade II and Clade IV, representing human *S. stercoralis* (Lineage A).

**Table 2:** Clinical data regarding the positive Strongyloidiasis patients using microscopic, serological, and molecular results.

ID	Group	Sex	Age	Residence	Symptoms	Microscopy	nPCR	ELISA
SR12	HIV+	Male	47	Rural	Abdominal pain and Diarrhea	+	+	+
ST27	Cancer (Lymphoma)	Male	32	Rural	Abdominal Pain	−	+	+
SS29	HIV+	Female	56	Rural	Abdominal Pain	+	+	+
SN61	Cancer (Breast)	Male	61	Urban	Diarrhea	+	+	+

## 4 Discussion

Strongyloidiasis is recognized as one of the most neglected infections among the neglected tropical diseases (NTDs) (Gordon et al., 2024). Numerous studies have demonstrated that the dormant state of *S. stercoralis* can persist for extended periods without leading to clinical signs (Rasul et al., 2021). The manifestation of clinical symptoms is often linked to a decrease in host immunity, which may result from debilitating illnesses, malnutrition, or the use of immunosuppressive drugs (Buonfrate et al., 2021). Immunocompromised patients who have lived in areas where strongyloidiasis is common may still face a risk of severe infection from *S. stercoralis* (Luvira et al., 2022). This risk exists even if they haven't been exposed to the infection for many years. When the immune system is weak, it can allow a dormant infection to become active again, which may have been present for a long time due to cycles of self-infection (Vasquez-Rios et al., 2019). Employing multiple diagnostic techniques on a single sample can enhance parasite detection since mixed methods exhibit varying sensitivities for different parasite species (Miswan et al., 2022). It has been reported that combining morphological characteristics with molecular methods can improve the sensitivity of diagnosing strongyloidiasis and provide a definitive diagnosis in clinic (Wang et al., 2017). In this study, 3.26 % of immunocompromised patients tested positive using wet mount slides, but it is noteworthy that the stool samples from these patients tested negative in agar plate cultures. The identification of rhabditiform larvae in stool samples is crucial for diagnosing *S. stercoralis* infections, as these larvae are indicative of the organism's presence in the gastrointestinal tract (Watts et al., 2016). Despite detecting these larvae microscopically, the negative results from agar plate cultures suggest that the larvae may not have developed into infective forms or that their numbers were insufficient for culture. The parasitological tests are the standard laboratory methods used to diagnose Strongyloides infections (Requena-Méndez et al., 2013). However, these tests have several limitations, including the irregular excretion of larvae, the requirement for multiple fresh stool samples, the necessity of having live larvae in culture media, and the challenge of distinguishing Strongyloides from similar nematodes. In addition, the use of certain drugs prior to sampling can significantly impact the results and complicate the diagnosis of parasitic infections (Ketzis, 2017).

Most published research on the diagnosis of human strongyloidiasis in immunocompromised individuals relies on serological and epidemiological investigation, indicating that serological methods can be effective screening tests for these populations (Kalantari et al., 2020; Weitzel et al., 2024). Current results of serological test conducted on samples from immunocompromised patients indicated infection rates of 20.65 %. Our results are in agreement with study conducted by Rafiei et al. (2016), which showed the sero-prevalence of *S. stercoralis* among immunocompromised patients in southwest Iran to be 14.4 % (Rafiei et al., 2016). In a systematic review and meta-analysis study (2022) in Iran, finding indicated that pooled prevalence of in immunocompromised patients utilizing serology, culture and microscopic methods was 10 % (95 % CI: 2 to 23), 1 % (95 % CI: 0 to 6) and 1 % (95 % CI: 0 to 1), respectively (Eslahi et al., 2022).

Data analysis revealed that the infection rate was significantly higher among cancer patients compared to HIV-positive individuals. The lower levels of IgG against Strongyloides in HIV-positive individuals, compared to cancer patients, can be attributed to several factors. AIDS is characterized by immune system dysfunction due to HIV infection, which leads to the destruction of CD4+ T cells and compromises the immune response (Okoye and Picker, 2013). This impairment disrupts the production of antibodies, including IgG, against pathogens such as Strongyloides. Furthermore, treatment regimens, such as antiretroviral therapy, may also influence antibody production in HIV-positive individuals (Okoye and Picker, 2013).

Both serological and molecular methods offer significantly higher sensitivity and faster results in diagnosing infections compared to traditional parasitological methods. However, each of these techniques has its limitations (Eslahi et al., 2022). It is important to note that serological tests may not always detect current infections, as antibodies can persist in the blood for months or even years after the infection has resolved. Therefore, serological tests should be used in simultaneous with other diagnostic methods to confirm the presence of *S. stercoralis* infection. Also, one major limitation of the ELISA method is the potential for immunological cross-reactions with other helminthic infections (Couturier and Theel, 2024).

The molecular analysis of the present study showed that 4. 35 % of stool samples had the DNA of the *S. stercoralis*. In recent years, molecular phylogenetic approaches utilizing mitochondrial and genomic markers have been employed to investigate the evolution and phylogeographic gene flow of *S. stercoralis* across various endemic regions worldwide (Fadaei Tehrani et al., 2019). Nagayasu et al. (2017) identified two distinct genetic lineages of *S. stercoralis*, including lineage A and lineage B. Lineage A is found in isolates from both humans and dogs in different countries, while lineage B is exclusively associated with dog isolates (Nagayasu et al., 2017). In another study, Sputin et al. (2019). reported that most isolates from several Asian countries can be categorized into these two lineages. They used cox1 gene marker, to accurately determine the taxonomic status and phylogeny of this parasite. Their analysis revealed two lineages: lineage A, comprising human isolates, and lineage B, consisting of isolates from canids (Spotin et al., 2019). Furthermore, lineage A was subdivided into four clades, including I, II, III, IV. Our phylogenetic analysis corroborated the microscopic diagnosis of the studied samples, showing that 75 % of the samples isolated in this study belonged to Clade IV, while 25 % were classified in Clade I, which belonged to human isolates. One of the limitation of this study was the small sample size, which comprised a total of 92 patients. The research was specifically conducted among immunocompromised individuals, a demographic that inherently presents challenges in recruitment due to the limited patient population in the region at the time of the investigation.

## 5 Conclusion

The study reveals the diagnostic challenges of *S. stercoralis* infections, as traditional methods like agar plate cultures and stool microscopy often yield negative results. In contrast, molecular techniques such as nested PCR effectively identified the parasite's DNA, particularly in high-risk groups like HIV-positive and cancer patients. Phylogenetic analysis indicated a predominance of human-associated Clade IV isolates, highlighting the need for improved diagnostic strategies. These results underscore the importance of employing a mixed diagnostic approach, combining parasitological, serological, and molecular techniques, to accurately identify infections in high risk populations. Given the potential for severe complications associated with strongyloidiasis in immunocompromised individuals, regular screening and prompt treatment are essential to reduce health risks. Furthermore, public health policy should prioritize the implementation of molecular diagnostic techniques in reference laboratories to enhance the accuracy of epidemiological surveillance and identify prevalent S. stercoralis strains.

## CRediT authorship contribution statement

**Reza Saberi:** Writing – original draft. **Aliasghar Ghorbanzadeh:** Investigation, Data curation. **Rabeeh Tabaripour:** Investigation. **Shahabeddin Sarvi:** Validation. **Shirzad Gholami:** Validation. **Seyed Abdollah Hosseini:** Writing – review & editing, Formal analysis.

## Declaration of competing interest

The authors declare no competing interests.
